# Acute Chest Syndrome: A Bibliometric Analysis of the Top 100 Most Cited Articles

**DOI:** 10.7759/cureus.52545

**Published:** 2024-01-19

**Authors:** Hassan Albarbari, Hashim M Al-awami, Hassan Aldibil, Ali Bazroon, Ali Almajid

**Affiliations:** 1 Department of Internal Medicine, King Fahad Specialist Hospital, Dammam, SAU; 2 Department of Internal Medicine, King Fahad University Hospital, Al Khobar, SAU

**Keywords:** sickle cell anemia, publication analysis, bibliometric analysis, sickle cell disease, acute chest syndrome

## Abstract

Acute chest syndrome (ACS) is a major cause of morbidity and mortality in patients with SCD (SCD). The analysis of research productivity and trends in ACS may serve as a valuable guide for investigators, institutions, and funding agencies to plan the future directions of research. The current review aims to evaluate the productivity and trends of publications related to ACS in adults by analyzing the top 100 most cited articles. A bibliometric analysis of the top 100 most cited articles related to ACS in adults was conducted on May 20, 2021. The Scopus database was searched to identify the top-cited articles. The following term was applied: “acute chest syndrome” in the fields of title, abstract, and keyword. The most cited article received a total of 776 citations, while the least cited received a total of 10 citations. Over half of the identified articles received 35 citations or less. The articles originated in 12 different countries; the overwhelming majority of articles originated in the United States (n = 75), with small contributions from developing countries with a high prevalence of sickle cell disease. Blood and American Journal of Hematology published the largest number of articles, with nine articles each. The Author “Vichinsky, E.P.” has the largest contribution with a total of 10 articles. The plethora of the highly cited articles were Observational studies, while randomized controlled trials were represented by seven articles. The present study demonstrates that research in ACS may be receiving less attention than it should. Therefore, research empowerment and adequate funding are of paramount importance to improve research productivity and quality. Additionally, more collaborative efforts should be encouraged to reduce the gap between developed and developing countries.

## Introduction and background

Among patients with SCD, acute chest syndrome (ACS) is a leading cause of death [1]. It is characterized by new radiodensity on chest radiographs along with respiratory symptoms and/or fever [2]. According to the Cooperative Study of Sickle Cell Disease (CSSCD), about half of patients with SCD, including both adults and children, experienced an episode of ACS [3]. Adults tend to have greater disease severity and higher mortality than children [4]. The higher mortality among adult patients is attributed to the increased incidence of fat emboli [4,5]. Therefore, prompt management, more research, and robust clinical trials are warranted to enhance the outcomes of patients with ACS.

Bibliometric analysis is a statistical method employed to assess productivity and trends in a particular area of research. Bibliometric analyses serve as valuable guides for investigators, institutions, and funding agencies to plan the future direction of research. There are limited analyses that have assessed the productivity and trends in SCD, including its complications. Recently, Musa et al. evaluated the global productivity of research on SCD between 1900 and 2020 using the Web of Science [6]. A total of 11,917 documents were retrieved, with an average of 27.34 citations for each document [6]. Similarly, Okoroiwu et al. investigated the productivity and trends of SCD research published over two decades (1997-2017) using the Scopus database [7]. A total of 19,921 documents were retrieved [7]. The review demonstrated that there is an exponential growth of scientific output on SCD, indicating a growing interest in this area of research [7]. To our knowledge, analyses concerning specific complications of SCD, including ACS, have not been conducted before. Therefore, the current review aims to evaluate the productivity and trends of publications related to ACS in adults by analyzing the top 100 most cited articles.

## Review

Search strategy

A bibliometric analysis of the top 100 most cited articles related to ACS in adults was conducted on May 20, 2021. The Scopus database was searched to identify the top-cited articles. The following term was applied: “acute chest syndrome” in the fields of title, abstract, and keyword (TITLE-ABS-KEY("acute chest syndrome")). The search yielded a total of 1,976 results, which were sorted based on the highest citation. No time restriction or any other filters were applied. Subsequently, titles, abstracts, and full texts were screened by two independent reviewers to ensure the relevance and eligibility of included publications. Each reviewer created a list of the top 100 most cited articles. Eventually, any discrepancies between the two lists were resolved through discussion.

Inclusion Criteria

This review aimed to explore the top 100 most cited articles written about ACS in adults. Original articles (clinical or basic) were considered; therefore, textbooks, review papers, abstracts, and case reports were excluded. Articles primarily investigating, but not necessarily exclusively, ACS were considered. The primary area of investigation was determined by screening introductions and objectives, where the primary focus should be clearly elaborated. The adult age group was the primary focus of this review; therefore, with basic science studies being an exception, any article involving adult subjects (18 years or older) was considered. In summary, the characteristics of eligible articles were as follows: (1) Original articles, (2) primarily investigating ACS, (3) involving adult subjects (18 years or older), and (4) Ranked as one of the top 100 most cited articles.

The citation count is influenced by many factors, including the passage of time. Subsequently, the current review may exclude relatively recent articles that may have a significant impact. Therefore, a second screening was carried out to extract the recent top 20 most cited articles. The results were filtered by year, and the search was limited to articles published between 2016 and 2021. Additionally, only original articles were considered, potentially posing a limitation in identifying a number of highly cited review papers or guidelines. Therefore, the top 10 highly cited systematic reviews or guidelines were extracted. No filter was applied at this stage.

Variables

The following variables were extracted: Year of publication, total citation, citation excluding self-citation, average citation per year, highest citation year, publishing journal, journal’s CiteScore, country affiliation (considered the first author’s affiliation), general study design (observational or experimental), specific study design (cross-sectional, case-control, cohort, randomized control trials, quasi-randomized, or basic research), funding (funded, non-funded, not mentioned), sample size (≤100, >100 - ≤ 300, > 300 - ≤ 600, or > 600). Additionally, the number of investigators, the first author's name, gender, h-index, and the number of contributed articles (from the top 100 most cited) were extracted. The variables were extracted manually and managed in an Excel sheet.

Statistical Analysis

The data were coded and analyzed using the Statistical Package for the Social Sciences, version 26.0 (IBM Corp., Armonk, NY, USA). Descriptive statistics were used to describe the productivity and trends of included articles. The years of publication were grouped into five-year intervals. The average citation per year was rounded to the nearest whole number.

Inferential statistics were run to evaluate the relationship between different variables. Pearson’s correlation coefficients were calculated to assess the correlation between the journals’ CiteScore and the number of published articles and total citations received by each journal, respectively. Furthermore, Pearson’s correlation coefficient was calculated to assess the correlation between the number of investigators and total citations. A Kendall’s tau-b test was employed to evaluate ranking discrepancies of included articles after disregarding self-citations. A Kruskal-Wallis H test was employed to assess the relationship between citation and study design.

Results

Citation Trends

The top 100 most cited articles are shown in Table 1. The most cited article received a total of 776 citations, while the least cited received a total of 10 citations. Concordantly, the most cited article received the highest rate of citations (35 per year). Kendall’s tau-b test demonstrates a very strong, positive correlation between the rankings of articles, including and excluding self-citations (τb = 0.910, p < 0.00).

**Table 1:** The top 100 most cited articles in ACS research TC: Total citations; ESC: citations excluding self-citations; C/Y: citation per year; HCY: highest citation year.

Rank	Article	TC	ESC	C/Y	HCY
1	Causes and outcomes of the acute chest syndrome in sickle cell disease	776	727	35	2016
2	The acute chest syndrome in sickle cell disease: Incidence and risk factors	561	552	21	2016
3	Acute chest syndrome in sickle cell disease: Clinical presentation and course	413	389	17	2014
4	Incentive spirometry to prevent acute pulmonary complications in sickle cell diseases	205	203	8	2011
5	Pulmonary fat embolism: A distinct cause of severe acute chest syndrome in sickle cell anemia	177	168	7	1999
6	Beneficial effect of intravenous dexamethasone in children	168	164	8	2011
7	Patterns of arginine and nitric oxide in patients with sickle cell	151	123	6	2004
8	Extracellular hemin crisis triggers acute chest syndrome in sickle mice	146	132	17	2016
9	Sickle cell acute chest syndrome: Pathogenesis and rationale for treatment	145	140	7	2004
10	Phospholipase A2 levels in acute chest syndrome of sickle cell disease	137	118	5	2009
11	'Acute Chest Syndrome' in Adults With Sickle Cell Anemia: Microbiology, Treatment, and Prevention	127	127	4	2001
12	Acute chest syndrome in sickle-cell disease	105	98	4	2005
13	Pulmonary hypertension and cor pulmonale during severe acute chest syndrome in sickle cell disease	1041	90	7	2012
14	Acute chest syndrome in sickle cell disease: Etiology and clinical correlates	92	92	3	1996
15	Pulmonary function in sickle cell disease with or without acute chest syndrome	91	88	4	2006
16	Acute chest syndrome in children with sickle cell disease: A retrospective analysis of 100 hospitalized cases	91	86	3	2005
17	Bronchoalveolar lavage in adult sickle cell patients with acute chest syndrome	89	75	3	2001
18	Acute chest syndrome in adults with sickle cell disease: Therapeutic approach	86	64	4	2005
19	Endothelin-1 production during the acute chest syndrome in sickle cell disease	83	78	4	2003
20	Asthma and acute chest in sickle-cell disease	79	56	4	2007
21	Secretory phospholipase A2 predicts impending acute chest syndrome in sickle cell disease	71	64	4	2009
22	Pulmonary artery thrombosis during acute chest syndrome in sickle cell disease	69	53	6	2018
23	Rib infarcts and acute chest syndrome in sickle cell diseases	68	65	3	1999
24	Association of T-786C eNOS gene polymorphism	67	67	4	2006
25	Clinical correlates of steady -state oxyhaemoglobin desaturation in children who have sickle cell disease	63	59	4	2014
26	Asthma is a risk factor for acute chest syndrome and cerebral vascular accidents in children with sickle cell disease	63	63	4	2007
27	Corticosteroids and increased risk of readmission after acute chest syndrome in children with sickle cell disease	61	58	5	2011
28	Pulmonary function abnormalities in childhood sickle cell disease	59	59	3	2008
29	Transfusion prevents acute chest syndrome predicted by elevated secretory phospholipase A2	56	50	4	2009
30	Opioid selection during sickle cell pain crisis and its impact on the development of acute chest syndrome	56	56	4	2008
31	Acute chest syndrome after abdominal surgery in children with sickle cell disease: Is a laparoscopic approach better?	55	53	3	2006
32	Plasma thrombospondin-1 is increased during acute sickle cell vaso-occlusive events	51	34	4	2015
33	Low exhaled nitric oxide and a polymorphism in the NOSI gene is associated with acute chest syndrome	51	46	3	2003
34	Plastic bronchitis and the role of bronchoscopy in the acute chest syndrome of sickle cell disease	51	50	3	2007
35	Mycoplasma disease and acute chest syndrome in sickle cell disease	49	49	3	2008
36	Association between plasma free haem and incidence of vaso-occlusive	47	43	6	2016
37	Safety of purified poloxamer 188 in sickle cell disease	46	39	3	2011
38	The impact of recurrent acute chest syndrome on the lung function of young adults with sickle cell disease	40	35	4	2017
39	Lower airway obstruction is associated with increased morbidity in children with sickle cell disease	40	31	3	2016
40	Clinician assessment for acute chest syndrome in febrile patients with sickle cell disease: Is it accurate enough?	40	33	2	216
41	Endothelial cell nitric oxide production in acute chest syndrome	40	28	2	2002
42	Simultaneous occurrence of rib infarction and pulmonary infiltrates in sickle cell disease	40	37	2	2000
43	Refining the value of secretory phospholipase A 2 as a predictor of acute chest syndrome	38	32	4	2016
44	Increased f2 isoprostanes in the acute chest syndrome of sickle cell disease as a marker of oxidative stress	37	33	2	2007
45	Elevated serum and bronchoalveolar lavage fluid levels of interleukin 8 and granulocyte	37	33	2	2004
46	Results of bronchoscopically obtained lower airway cultures from adult sickle cell	37	34	2	2001
47	The diagnosis of pulmonary thromboembolism in sickle cell disease	36	35	1	1999
48	Acute kidney injury in sickle patients with painful crisis or acute chest syndrome	35	28	3	2017
49	Exchange versus simple transfusion for acute chest syndrome in sickle cell anemia adults	35	35	3	2013
50	Decreased exhaled nitric oxide in sickle cell disease: Relationship with chronic lung involvement	35	35	2	2006
51	Chlamydia pneumoniae and acute chest syndrome in patients with sickle cell disease	35	33	2	2005
52	Chronic pulmonary disorders in sickle cell disease: Findings at thin- section CT	35	35	2	2003
53	Mortality, asthma, smoking and acute chest syndrome in young adults with sickle cell disease	34	32	4	2016
54	Effect of Red Cell Exchange Transfusion on Plasma Levels of Inflammatory Mediators	33	33	3	2008
55	Factors predicting future ACS episodes in children with sickle cell anemia	32	22	4	2017
56	Clinical presentation of acute chest syndrome in sickle cell disease	30	30	2	2007
57	Risk factors for acute chest syndrome in children with sickle cell disease undergoing abdominal surgery	30	30	2	2008
58	Induced Sputum versus Bronchoalveolar Lavage during Acute Chest Syndrome in Sickle Cell Disease	30	22	2	2009
59	Erythrocytapheresis in children with sickle cell disease and acute chest syndrome	29	27	3	2013
60	Acute chest syndrome in adult Afro-Caribbean patients with sickle cell disease	29	29	2	2015
61	Elevated Plasma sVCAM-1 Levels in Children With Sickle Cell Disease	28	23	2	2005
62	Early intermittent noninvasive ventilation for acute chest syndrome in adults	27	20	2	2012
63	Steady-state sVCAM-1 serum levels in adults with sickle cell disease	27	17	1	2008
64	Pulmonary function and airway hyperresponsiveness in adults with sickle cell disease	26	26	3	2011
65	Tapered oral dexamethasone for the acute chest syndrome of sickle cell disease	25	19	2	2018
66	Secretory phospholipase A2 levels in patients with sickle cell disease and acute chest syndrome	25	20	2	2012
67	Smoking is a factor in causing acute chest syndrome in sickle cell anemia	25	24	1	2013
68	Physician-diagnosed asthma and acute chest syndrome: Associations with NOS polymorphisms	24	21	2	2009
69	Pulmonary function abnormalities and asthma are prevalent in children with sickle cell disease and are associated with acute chest syndrome	22	22	2	2013
70	Multi-modal intervention for the inpatient management of sickle cell	22	20	2	2017
71	The use of bilevel positive airway pressure for the treatment of acute chest syndrome of sickle cell disease	22	22	2	2011
72	Platelet extracellular vesicles drive inflammasome-IL-1β-dependent lung injury in sickle cell disease	21	16	16	2020
73	Smoking is associated with an increased risk of acute chest syndrome and pain among adults with sickle cell disease	21	17	2	2013
74	Lung imaging during acute chest syndrome in sickle cell disease	21	16	3	2020
75	Hemin causes lung microvascular endothelial barrier dysfunction by necroptotic cell death	20	20	5	2021
76	Pulmonary platelet thrombi and vascular pathology in acute chest syndrome	20	20	4	2019
77	A nontransfusional perioperative management regimen for patients	20	18	2	2009
78	Vitamin D deficiency and acute vaso-occlusive complications in children with sickle cell disease	19	17	3	2018
79	Outcomes of acute chest syndrome in adult patients with sickle cell disease: Predictors of mortality	19	19	3	2019
80	Gene-centric association study of acute chest syndrome and painful crisis in sickle cell disease patients	19	18	3	2016
81	Effect of oral arginine supplementation on exhaled nitric oxide concentration in sickle cell	19	19	5	2014
82	Sickle cell disease patients in eastern province of Saudi Arabia suffer less severe acute chest	19	19	2	2011
83	Rapidly progressive acute chest syndrome in individuals with sickle cell anemia	18	16	4	2018
84	Inhaled nitric oxide for acute chest syndrome in adult sickle cell patients: a randomized controlled study	18	16	3	2019
85	Clinical factors and incidence of acute chest syndrome or pneumonia among children	18	18	3	2016
86	A short course of prednisone in the management of acute chest syndrome of sickle cell disease	18	17	2	2011
87	Urinary cysteinyl leukotriene e 4 is associated with increased risk for pain	18	14	2	2011
88	Comparison of automated red cell exchange transfusion and simple transfusion	16	16	2	2020
89	Acute chest syndrome is associated with history of asthma in hemoglobin SC disease	16	16	2	2013
90	Serum biomarkers for identifying acute chest syndrome	16	16	1	2012
91	Sera of patients suffering from inflammatory diseases contain group IIA	16	16	1	2004
92	Acute chest syndrome of sickle cell disease: Radiographic and clinical analysis of 70 cases	16	16	1	1999
93	Quantitative intravital two-photon excitation microscopy	16	9	2	2020
94	Association of plasma CD40L with acute chest syndrome in sickle cell anemia	15	13	4	2020
95	A single-institution experience with treatment of severe acute chest syndrome	15	15	2	2011
96	Hematologic changes during acute chest syndrome in sickle cell disease	13	12	1	2002
97	Score Predicting Acute Chest Syndrome During Vasoocclusive Crises in Adult	12	8	2	2018
98	Cholecystectomy in sickle cell disease patients: Is there more acute chest syndrome after laparoscopy?	11	11	1	2009
99	Acute chest syndrome among children hospitalized with vaso-occlusive crisis	10	7	3	2019
100	Exosomes contribute to endothelial integrity and acute chest syndrome risk: Preliminary findings	10	7	2	2020

Countries and Years

**Figure 1:**
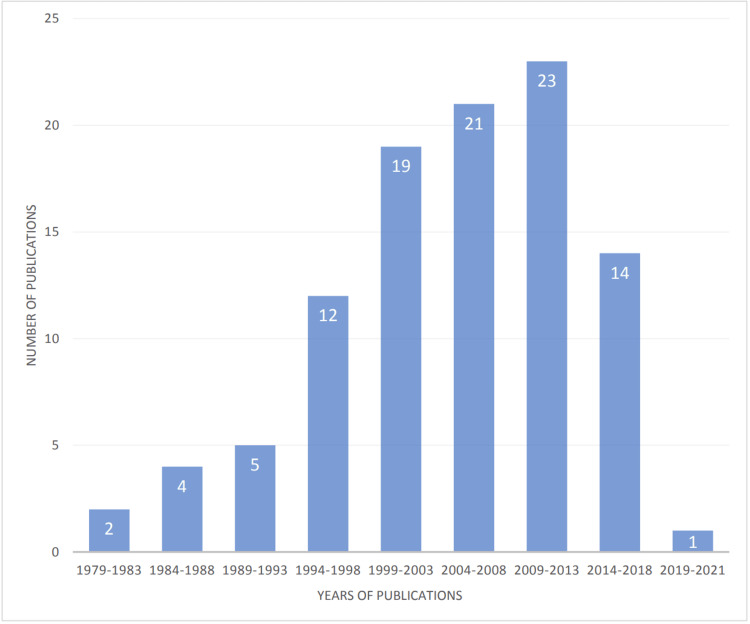
Year distribution of the top 100 most cited articles in ACS research

The articles originated in 12 different countries, as shown in Table 2. Nevertheless, the overwhelming majority of articles originated in the United States (n = 75). The publication year distribution was grouped into five-year windows, as shown in Figure 1. There was an upward trend in the number of highly cited articles as a function of the year. About a quarter of the top 100 cited articles were published between 2009 and 2013.

**Table 2:** Country distribution of the top 100 most cited article in ACS TC: Total citations; TP: Total publication

Country	TP	TC
United States	75	5281
United Kingdom	4	223
France	11	582
Canada	1	55
Jamaica	2	74
Curaçao	1	27
Turkey	1	26
Brazil	1	15
Martinique	1	11
Saudi Arabia	1	19
Netherlands	1	29
Lebanon	1	37

Journals

The top 20 most cited journals are shown in Table 3. A total of 45 journals were identified. Blood and the American Journal of Hematology published the largest number of articles, with nine articles each. Additionally, the highest citations were received by Blood (1,712), followed by the New England Journal of Medicine (981) and the American Journal of Respiratory and Critical Care Medicine (484). The correlation between the CiteScore and the number of published articles was insignificant (r = 0.72, p = 0.646). However, the CiteScore and total citations were found to be moderately positively correlated (r = 0.482, p = 0.001).

**Table 3:** The top 20 most cited journal in ACS TC: Total citations; TP: Total publication

Journal	TP	TC
Blood	9	1712
New England Journal of Medicine	2	981
American Journal of Respiratory and Critical Care Medicine	8	484
Journal of Pediatric Hematology Oncology	7	342
British Journal of Haematology	7	333
American Journal of Hematology	9	271
Pediatric Blood and Cancer	8	229
The Lancet	2	173
Archives of Internal Medicine	2	156
Pediatric Pulmonology	4	153
The Journal of Pediatrics	2	151
Journal of Clinical Investigation	1	146
Chest	2	137
Lung	3	100
European Respiratory Journal	1	91
Journal of Pediatric Surgery	2	85
Hemoglobin	2	71
Pediatrics	2	65
Clinical and Molecular Allergy	1	63
Intensive Care Medicine	2	45
Annals of Emergency Medicine	1	40

Investigators

The top 20 contributing first authors are listed in Table 4. “Vichinsky, E.P.” has the largest contribution with a total of 10 contributed articles. Concordantly, he secured the highest h-index (81). The number of investigators and average citation per year were found to be weakly positively correlated (r = 0.330, p = 0.001). The majority of research was funded (n = 60), while a minority was not (4); the funding was not mentioned in the rest of the articles (n = 36).

**Table 4:** The top 20 most contributing authors in ACS research

Name	Gender	h-index	Total contributions	Contributions as first author
Vichinsky, E.P.	M	81	10	3
Godeau, B.	M	59	9	1
Styles, L.A.	F	38	8	4
Maitre, B.	M	34	8	1
Ballas, S.K.	M	50	7	1
DeBaun, M.R.	M	55	7	1
Dessap, A.M.	M	21	5	2
Abboud, M.R.	M	42	5	1
Rucknagel, D.L	M	26	4	1
Castro, O.	M	47	3	1
Quinn, C.T.	M	30	3	1
Neumayr, L.	F	24	3	1
Duckworth, L.	M	13	3	1
Morris, C.R.	F	30	2	2
Ghosh, S.	M	19	2	1
Stuart, M.J.	F	38	2	1
Davies, S.C.	F	36	2	1
Poncz, M.	M	62	2	1
Hammerman, S.I	M	3	2	2
Boyd, J.H.	F	10	2	2

Study Designs

The study designs are shown in Table 5. Observational studies constitute the vast majority of the conducted (n = 85) and cited studies (n = 5,438), with cohort study being the most frequently employed study design (n = 43). On the contrary, experimental studies constituted a minor fraction (n = 15), with RCT being only represented by seven studies. A Kruskal-Wallis H test showed that total citations were not affected by study design (H = 5.926, p = 0.548). The sample size categories are shown in Table 6. The majority of the studies involved a sample size of 100 participants or fewer (n = 62). On the other hand, only a total of five studies included a sample size of more than 600 participants.

**Table 5:** Study designs of the top 100 most cited articles in ACS research TC: Total citations; TP: Total publication; RCT: Randomized-controlled trial

Study design	TP	TC
Cross sectional	22	1829
Case control	20	1196
Cohort study	43	2413
RCT	7	527
quasi-RCT	1	56
Basic research	7	358

**Table 6:** Sample sizes of the most cited study TC: Total citations; TP: Total publication

Sample size	TP	TC
≤ 100	62	3073
> 100 - ≤ 300	22	1005
> 300 - ≤ 600	8	1030
> 600	5	1089

Recent and Review Publications

The top 20 most recently cited articles are listed in Table 7. The most cited recent article received 21 citations, while the least cited received four citations. A total of 12 studies were not identified in the initial list of the top 100 most cited articles. The top 10 most cited systematic reviews and guidelines are listed in Table 8. This list represents all identified systematic reviews and guidelines found by our search. Two of the identified review papers have not received any citations.

**Table 7:** The top 20 recent most cited articles in ACS research TC: Total citations

Rank	Article	TC
1	Platelet extracellular vesicles drive inflammasome-IL-1βdependent lung injury in sickle cell disease	21
2	Hemin causes lung microvascular endothelial barrier dysfunction by necroptotic cell death	20
3	Pulmonary platelet thrombi and vascular pathology in acute chest syndrome in patients with sickle cell disease	20
4	Rapidly progressive acute chest syndrome in individuals with sickle cell anemia: a distinct acute chest syndrome phenotype	18
5	Association of plasma CD40L with acute chest syndrome in sickle cell anemia	15
6	Score Predicting Acute Chest Syndrome During Vaso-occlusive Crises in Adult Sickle-cell Disease Patients	12
7	Acute chest syndrome among children hospitalized with vasoocclusive crisis: A nationwide study in the United States	10
8	Exosomes contribute to endothelial integrity and acute chest syndrome risk: Preliminary findings	10
9	Bedside lung ultrasound during acute chest syndrome in sickle cell Disease	9
10	Extracorporeal Life Support for Severe Acute Chest Syndrome in Adult Sickle Cell Disease: A Preliminary Report	8
11	Augmented NRF2 activation protects adult sickle mice from lethal acute chest syndrome	8
12	Association of guideline-adherent antibiotic treatment with readmission of children with sickle cell disease hospitalized with acute chest syndrome	8
13	Exhaled nitric oxide: Not associated with asthma, symptoms, or spirometry in children with sickle cell anemia	8
14	Pulmonary vascular dysfunction and cor pulmonale during acute respiratory distress syndrome in sicklers	8
15	Accuracy of Point-of-care Lung Ultrasonography for Diagnosis of Acute Chest Syndrome in Pediatric Patients with Sickle Cell Disease and Fever	7
16	Original Research: Acute chest syndrome in sickle cell disease: Effect of genotype and asthma	7
17	Factors associated with mechanical ventilation use in children with sickle cell disease and acute chest syndrome	6
18	Aeroallergen sensitization predicts acute chest syndrome in children with sickle cell anaemia	5
19	Early noninvasive ventilation and nonroutine transfusion for acute chest syndrome in sickle cell disease in children: A descriptive study	4
20	Inflammatory and endothelial markers during vaso-occlusive crisis and acute chest syndrome in sickle cell disease	4

**Table 8:** The top 10 most cited guidelines and reviews in ACS TC: Total citations

Rank	Article	TC
1	Guideline on the management of acute chest syndrome in sickle cell disease	73
2	Inhaled nitric oxide for acute chest syndrome in people with sickle cell disease	16
3	Nitric oxide metabolism and the acute chest syndrome of sickle cell anemia	13
4	Vitamin D supplementation for sickle cell disease	9
5	Blood transfusions for treating acute chest syndrome in people with sickle cell disease	9
6	Inhaled bronchodilators for acute chest syndrome in people with sickle cell disease	5
7	Obstructive sleep apnea in sickle cell disease carriers | [Apneia obstrutiva do sono em portadores da anemia falciforme]	2
8	Antibiotics for treating acute chest syndrome in people with sickle cell disease	2
9	How I Treat Acute Chest Syndrome in Asthmatic Children with Sickle Cell Disease. A Practical Review	0
10	Respiratory therapy in children with sickle cell disease and acute chest syndrome | [Fisioterapia respiratória em crianças com doença falciforme e síndrome torácica aguda]	0

Discussion

Productivity

The highest number of citations has not exceeded a total of 776 citations. In a previous report, the highest-cited article related to SCD received a total of 7,507 citations [6]. Productivity in other areas of medicine is considerably larger; this suggests lower research activity and less interest in SCD and related complications [8-10]. Over half of the identified articles received 35 citations or less, further suggesting decreased productivity. Lower productivity may hinder the development of new and more effective treatments for ACS. One study compared the funding between SCD and cystic fibrosis, which has a lower prevalence, and the associated research productivity; the study highlighted funding disparities between SCD and cystic fibrosis, which might contribute to decreased research productivity and ancillary therapeutic invention [11]. Therefore, more efforts are warranted to enhance research productivity and the development of novel treatments for ACS.

Publications Trends

Millions of people around the world are affected by SCD. It is particularly prevalent in Central and South America, Saudi Arabia, India, and Mediterranean countries. For example, in Jamaica, one in every 150 persons is affected by SCD [12]. In Saudi Arabia, approximately one in every 260 persons has SCD [13]. Despite having a lower prevalence, the USA contributed the vast majority of highly cited articles in ACS, with minimal contributions from developing countries. Developing countries, especially economically disadvantaged nations, may be disproportionately affected by the burden of SCD complications. Therefore, the gap between developed and developing countries should be bridged, and more collaborative efforts should be encouraged.

The largest number of publications were published in “Blood” and “American Journal of Hematology,” with nine articles each. In concordance, previous bibliometric analyses identified these two journals as the most productive journals in SCD [6,7]. Hence, these journals could be recommended for investigators interested in this field. “Vichinsky, E.P.” (h-index = 81) has been identified as the most contributing author with a total of 10 articles. In agreement with our result, Okoroiwu et al. identified “Vichinsky, E.P.” as the most contributing author in SCD research; however, Musaa et al. identified “Serjeant GR” as the most productive author [6,7]. Highlighting the most contributing authors in ACS may help investigators around the world plan future directions and collaborations.

It has been noted that author self-citations account for one in 15 citations of articles published in high-profile general medicine journals [14]. Though the self-citation phenomenon may inflate the importance of some scientific literature, we found that self-citation has no significant impact on the final ranking of articles [15].

The plethora of highly cited articles was observational in design. Approximately half of those were cohort studies, which yield stronger evidence than other types of observational studies. On the other hand, we identified a paucity of RCTs, with only seven articles represented in the list. Generally, RCTs are the best study type for proving causality. Slightly below two-thirds of studies enrolled a sample size of less than 100. Notably, the power of a study, the probability of establishing a statistically significant result, is inherently related to the sample size [16]. Therefore, research empowerment and adequate funding are crucial to ensure the integrity of the literature.

Top Three Cited Articles

In 2000, the National Acute Chest Syndrome Study Group published the most cited articles related to ACS. The study was published in “The New England Journal of Medicine,” with “Vichinsky, E.P.” being the first author. In this multicenter study, which involved 30 centers, a total of 671 episodes of ACS were analyzed to determine the cause and outcome [4]. Interestingly, approximately half of the patients were initially hospitalized for another cause, such as a painful crisis [4]. The cause of ACS was found in 38% of all episodes. The most commonly identified causes were pulmonary fat embolism and infection; pulmonary emboli and bronchopneumonia were the most common causes of death [4]. Additionally, the study has demonstrated that phenotypically matched transfusions and the use of bronchodilators improved oxygenation and clinical outcomes. With aggressive management, most patients with respiratory failure will successfully recover [4]. Nevertheless, ACS was associated with a 3% mortality rate [4].

The year 1994 witnessed the publication of the second most cited article related to ACS by Castro et al., involving participants of CSSCD. In that study, a total of 3,751 patients with sickle cell disease (SCD), who were followed for at least two years, were examined to determine the incidence and risk factors of ACS [3]. The total number of events was 2,100. The incidence was highest among patients with homozygous (SS) SCD and lowest among patients with sickle cell-beta(+) thalassemia [3]. The incidence was significantly correlated with younger age groups. However, a higher mortality rate was observed in adults with a higher incidence rate [3]. Furthermore, they identified higher steady-state Hb levels and lower fetal Hb as independent risk factors for ACS among patients with SS disease [3].

In 1997, Vichinsky et al. published the third most cited article, where they examined the clinical presentation and course of ACS, analyzing the participants of CSSCD [17]. Adults commonly presented with dyspnea, chills, and severe pain, and they were often afebrile. Multilobe and lower lobe disease were commonly observed in adults [17]. Besides, mortality was four times higher in adults than in children. Rapid development of respiratory failure was generally seen in fatal cases [17].

Limitations

A number of limitations should be considered in this analysis. First, we searched a single database, which restricts the identification of highly cited articles. Second, many non-English articles might not be identified due to the possibility of English language bias in the utilized database. Third, textbooks and abstracts of conference proceedings are not included in the present analysis, and therefore, some of the highly cited publications may be omitted. Finally, the ranking of articles is based on citations, and the importance of an article should not be solely based on citation count. Nevertheless, citation count is still considered a valuable supplementary tool to assess the impact of a particular publication [18].

## Conclusions

ACS has been identified as a major cause of morbidity and mortality in patients with SCD. The present bibliometric analysis highlights the characteristics of the top 100 most-cited articles in ACS research. The study demonstrates that research on ACS is receiving less attention than it should, and this may impede the development of novel treatments. Increasing funding and support may be required to boost research productivity in this field. Furthermore, the vast majority of highly cited articles are generated in the US, with a small contribution from developing countries with a high prevalence of SCD. Therefore, more collaborative efforts should be encouraged to reduce the gap between developed and developing countries, ensuring that these disadvantaged populations are not disproportionately affected. Additionally, a paucity of highly cited RCTs is noted, and a considerable number of studies had a relatively small sample size. Therefore, research empowerment and adequate funding are of paramount importance to improve research productivity and quality. The present work may serve as guidance to investigators and funding agencies to plan the future direction of ACS research with hope that the future of research in this area becomes more fruitful. Exploring future directions could involve a concentrated effort on preventive interventions and innovative therapeutic options in ACS.
